# Development of Artificial Intelligence‐Supported Automatic Three‐Dimensional Surface Cephalometry

**DOI:** 10.1111/ocr.12914

**Published:** 2025-03-04

**Authors:** Chihiro Tanikawa, Hiroyuki Nakamura, Takaaki Mimura, Yume Uemura, Takashi Yamashiro

**Affiliations:** ^1^ Department of Orthodontics and Dentofacial Orthopedics, Graduate School of Dentistry Osaka University Suita Osaka Japan; ^2^ Technology Research & Innovation BIPROGY Incorporated Company Koto‐ku Tokyo Japan; ^3^ Technology Management Division UEL Corporation Koto‐ku Tokyo Japan

**Keywords:** artificial intelligence, cephalometry, humans, spiral cone‐beam computed tomography

## Abstract

**Objective:**

Surface‐based three‐dimensional (3D) cephalometry provides detailed clinical information for the analysis of craniofacial structures. This study aimed to develop an automated 3D surface cephalometry system using mesh fitting based on landmarks identified by artificial intelligence (AI) and to evaluate its accuracy.

**Methods:**

A total of 185 CBCT images from adult Japanese patients (system training, *n* = 152; evaluation, *n* = 33) were used in this study. Cranial and mandibular images were generated via surface rendering of CBCT images. An experienced orthodontist manually recognised 19 and 45 3D landmarks for the cranium and mandible, respectively, and used them as the gold standard after they were checked by another experienced orthodontist. An AI system developed using PointNet ++ was trained to output landmark coordinates based on surface data and normal vectors. Mesh fitting (homologous modelling) was then conducted using the AI‐identified landmarks. The errors in mesh fitting were evaluated.

**Results:**

The mean errors for wire mesh fittings with AI‐identified landmarks for the maxilla and mandible were 0.80 ± 0.57 mm and 1.45 ± 0.34 mm, respectively.

**Discussion:**

An AI‐based landmark identification system and mesh fittings that demonstrate clinically acceptable accuracy were presented. This system can be applied in clinical settings to quantify and visualise craniofacial structures in three dimensions.

**Conclusion:**

The automated 3D surface cephalometry system utilising mesh fitting based on AI‐identified landmarks showed clinically acceptable accuracy. This allows orthodontists to compare a patient's craniofacial surface with normative data, without the need for manual landmark identification.

## 1 Introduction

In orthodontic practice, objective and quantitative evaluation of craniofacial morphology is crucial for treatment planning, assessment, and prognosis. Cone‐beam computed tomography (CBCT) imaging provides high‐resolution three‐dimensional (3D) views of the maxillofacial skeleton, offering detailed visualisation of the anatomical structure. However, effectively quantifying the complex morphology captured in these images remains a challenge [1]. Traditional two‐dimensional (2D) cephalometric analyses or recently developed ‘3D cephalometry’ [1, 2, 3], which both rely on angular and linear measurements, are insufficient for comprehensively assessing the curved surfaces of craniofacial structures, thus highlighting a significant limitation in current methods.

To address these challenges, a novel 3D surface‐based approach was developed. In this approach, the maxillofacial shape is represented by points and connecting lines overlaying a template wire mesh with common landmarks [4]. This transforms individual shapes into a homologous model (or wire mesh model), enabling the comparison and generation of average images and the morphologies of individuals. Our initial application of this method in animal models successfully captured craniofacial changes due to dietary intervention, demonstrating its utility in morphological studies [4].

This 3D surface‐based approach was extended to human subjects by deriving normative craniofacial skeletons from CBCT images of adults with skeletal Class I occlusion [5]. Utilising these normative data, a ‘3D surface cephalometry’ system was established. This system can detect deviations in individual craniofacial morphology from established norms. Clinically, this system offers entire quantitative analysis of hard tissue morphology in regions where traditional landmarks are difficult to define, such as the lateral surface of the anatomical structure of the maxilla and mandible. For example, our previous study [5] showed that in a patient with Lowys‐Dietz syndrome, the mandibular body anterior to the antegonial notch and the mandibular angle were displaced backward and downward, whereas the condyle and coronoid process, except for its tip, were displaced forward and downward, which was difficult to evaluate using previous landmark‐based systems [1, 2, 3]. By combining the surface‐based approach with a principal component analysis, our system allows for the comprehensive evaluation of shape characteristics and individual classification. Importantly, sex‐based variations in craniofacial morphology were also statistically confirmed, further refining the accuracy of this method in clinical applications [6]. Three‐dimensional surface‐based approaches are anticipated to be powerful tools for planning, monitoring, and evaluating craniofacial morphology and growth, because they retain comprehensive information on the shape of the surfaces.

Despite the advances achieved with this 3D surface‐based approach, manually identifying landmarks on 3D surfaces is required as one of the whole steps and is a time‐consuming task, which limits the efficiency of this method. Automating this step would substantially improve efficiency and consistency. Leveraging AI, specifically deep learning algorithms, offers a promising solution for automatically detecting 3D landmarks with high precision. AI‐driven landmark identification not only accelerates the analyses but also reduces human error, making it highly suitable for clinical applications. Thus far, several recognition systems have been developed for 3D landmarks on 3D surfaces or CT voxel data [7, 8, 9, 10, 11, 12, 13, 14]. In particular, a recent voxel‐based 3D landmark recognition system [15] reported an error of < 2 mm for 32 landmarks and showed clinical significance as a landmark‐based 3D cephalometry system. Nonetheless, the clinical accuracy of automatic 3D ‘surface’ cephalometry following an AI‐supported 3D landmark recognition system has not been well described.

Therefore, the present study aimed to develop an automatic 3D surface cephalometry system following an AI‐supported 3D landmark recognition system and to demonstrate its accuracy in clinical settings.

## 2 Materials and Methods

Ethical approval for this study was obtained from the Institutional Review Board of Osaka University Dental Hospital (No. H30‐E5‐1). The requirement for informed consent was waived because of the retrospective nature of the study.

### 2.1 Dataset and Three‐Dimensional Reconstruction

A total of 185 CBCT images of pre‐treatment adult Japanese patients (system training, *n* = 152; evaluation, *n* = 33; age range, 18–35 years; ANB angle = 0.23° ± 2.84°) who visited a private clinic between April 2013 and March 2020 were consecutively investigated and included in this study. The inclusion criteria were an age range of 18–35 years old and availability of CBCT data with a field of view of 20 cm × 20 cm. The exclusion criteria were a history of trauma or injury to the face, congenital anomalies, significant abnormal bone defects (such as tumours), significant artefacts in the CBCT data, and absence of more than three teeth (except third molars).

CBCT was conducted using an Alphard‐3030 in the low‐dose mode (Asahi Roentgen Ind. Co. Ltd., Kyoto, Japan) at 80 kV and 2 mA with a 1‐voxel size of 0.39 mm. Low‐dose modes enable the acquisition of 3D images with less radiation exposure to patients than panoramic and cephalometric radiographs, while providing either an equivalent or better resolution and more information [16]. This includes details of fractured teeth, anatomical structures for the placement of implants, location of impacted teeth, resorption of adjacent teeth, and airway morphology [17]. In addition, CBCT data can be repurposed to generate 2D cephalograms and panoramic radiographs, eliminating the need for conventional X‐ray images and reducing overall radiation exposure [18]. In our cases, CBCT data was primarily used for the examination of the location of impacted teeth, resorption of adjacent teeth and surgical cases, as recommended in a previous study [17]. For these patients, the CBCT data were secondarily utilised to generate 2D frontal and lateral cephalograms as well as panoramic radiographs. This process requires a field of view of 20 × 20 cm, closely matching the dimensions of typical cephalometric film, which measures 20 × 25 cm [19]. Scans were performed only when the diagnostic benefits outweighed the risks to ensure patient safety.

**FIGURE 1:**
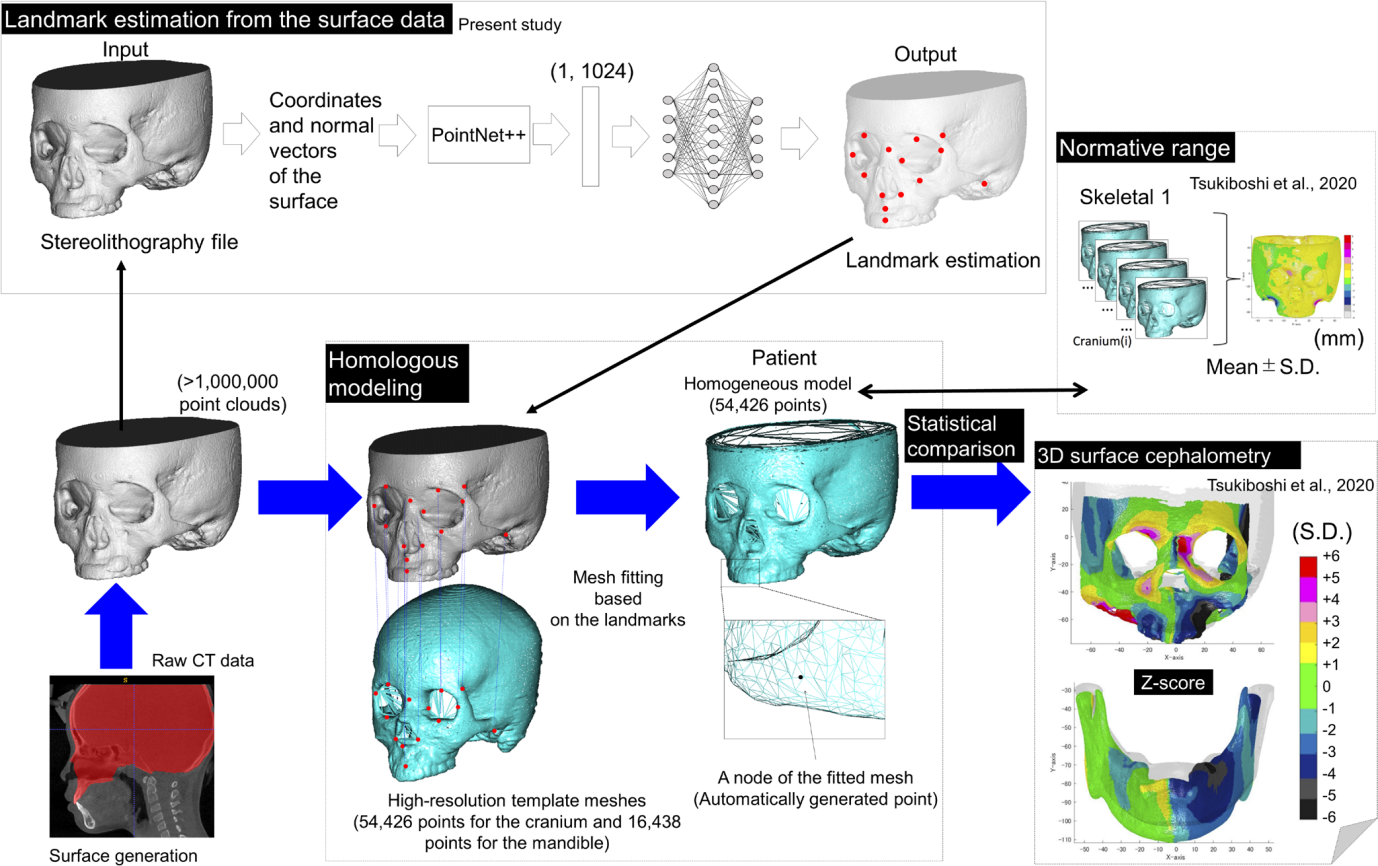
Schematic illustration demonstrating the wire mesh fittings based on the automatically identified landmarks and the application of the fitted mesh for a surface‐based 3D cephalometry system in the clinical setting.

Three‐dimensional reconstructions of the crania and mandible were obtained using the ITK‐SNAP open‐source software program (ITK‐SNAP, National Library of Medicine and National Institutes of Health, Bethesda, MD, USA; Figure 1). The software stores slices captured by CT scanners as digital imaging and communications in medicine (DICOM) images to generate 3‐D models corresponding to anatomical parts of the human body by allowing the conversion of a stack of DICOM files into a stereolithography file. After importing the DICOM files into the ITK‐SNAP software program, the cranial and mandibular surfaces were segmented using combined semi‐automatic and manual segmentation. One of the authors manually determined the threshold values (lower to higher) for each patient so that the cranial and mandibular shapes could be segmented according to the method outlined in a previous study [20] that manual threshold selection showed better surface models compared to the pre‐selected standardised threshold. Next, by combining the neighbouring voxels showing grayscale values ranging between the determined thresholds, the 2D areas inside the cranial and mandibular structures were determined for each slice image in the DICOM and Communications in Medicine files. If necessary, a ‘paintbrush’ tool for manual segmentation was used to remove areas not relevant to the structure of interest. The segmentation encompassed the entire cranium within the CBCT field of view, as well as the entire mandible. The series of areas determined for each slice image were combined as a 3D surface model, and the models of the cranium and mandible were exported as stl files. This process took approximately 30–60 min for each patient.

### 2.2 Manual Landmark Identification (Gold Standard) and Coordinate System

For the 3D model of each patient, an experienced orthodontist (YU) manually identified 19 and 45 3D landmarks [2, 21, 22, 23] for the cranium and mandible, respectively, using a mouse cursor and identification software program (HBM Rugle, Medical Engineering Inc., Kyoto, Japan) (Supporting Information S1). These landmarks were checked by another experienced orthodontist (CT; university faculty) and employed as the gold standard (GS) for system training and evaluation. The reliability of landmark identification has been previously reported (YU is the orthodontist who identified the landmarks in a previous study [5]), and all landmarks showed errors of < 2 mm. In our previous study [5], landmarks were selected to create a mesh for 3D surface cephalometry; thus, the present study employed only landmarks that were needed for mesh fittings and 3D surface cephalometry. In our previous study, the inclusion and exclusion of landmarks were described [5]. The selection of landmarks is detailed in Supporting Information S2 and illustrated in Figures [Link], [Link], [Link]. A previous study evaluated the intra‐ and inter‐examiner reliabilities of the mesh fitting method using these landmarks [5], demonstrating almost perfect [24] reliability.

After segmenting and identifying the landmarks, a system of coordinates for each 3D image was established based on the landmarks for each cranial and mandibular surface [5]. Nasion was defined as the origin of cranial and mandibular surfaces. The sagittal plane was defined as the plane passing through the origin and perpendicular to the line through the right and left zygomaticofrontal sutures, respectively. The axial plane was defined as the plane passing through the origin and parallel to the line connecting the midpoints of the bilateral porions and orbitales, representing the Frankfort horizontal plane. The coronal plane was defined as the plane passing through the origin, perpendicular to both the axial and sagittal planes. The coordinates of the landmarks were standardised using this coordinate system.

### 2.3 AI Systems

An AI system that outputs the coordinate values of landmarks based on a stereolithography file is developed using PointNet ++ [25]. The details are presented in Figure 1; Supporting Information S3.

### 2.4 Evaluation

The trained AI systems for each cranium and mandible were evaluated using the dataset stored for the system evaluation. Landmarks that were automatically identified by the AI system (AI) were compared with the GS as absolute differences in each axis (transverse [*x*‐axis], vertical [*y*‐axis], and anteroposterior [*z*‐axis]). Furthermore, the identification errors were expressed as 90% confidence ellipses. The confidence ellipse defines the region that contains 90% of all samples that can be drawn from the underlying Gaussian distribution [26].

### 2.5 Mesh Fitting Based on the AI‐Identified Landmarks and Its Accuracy

**FIGURE 2:**
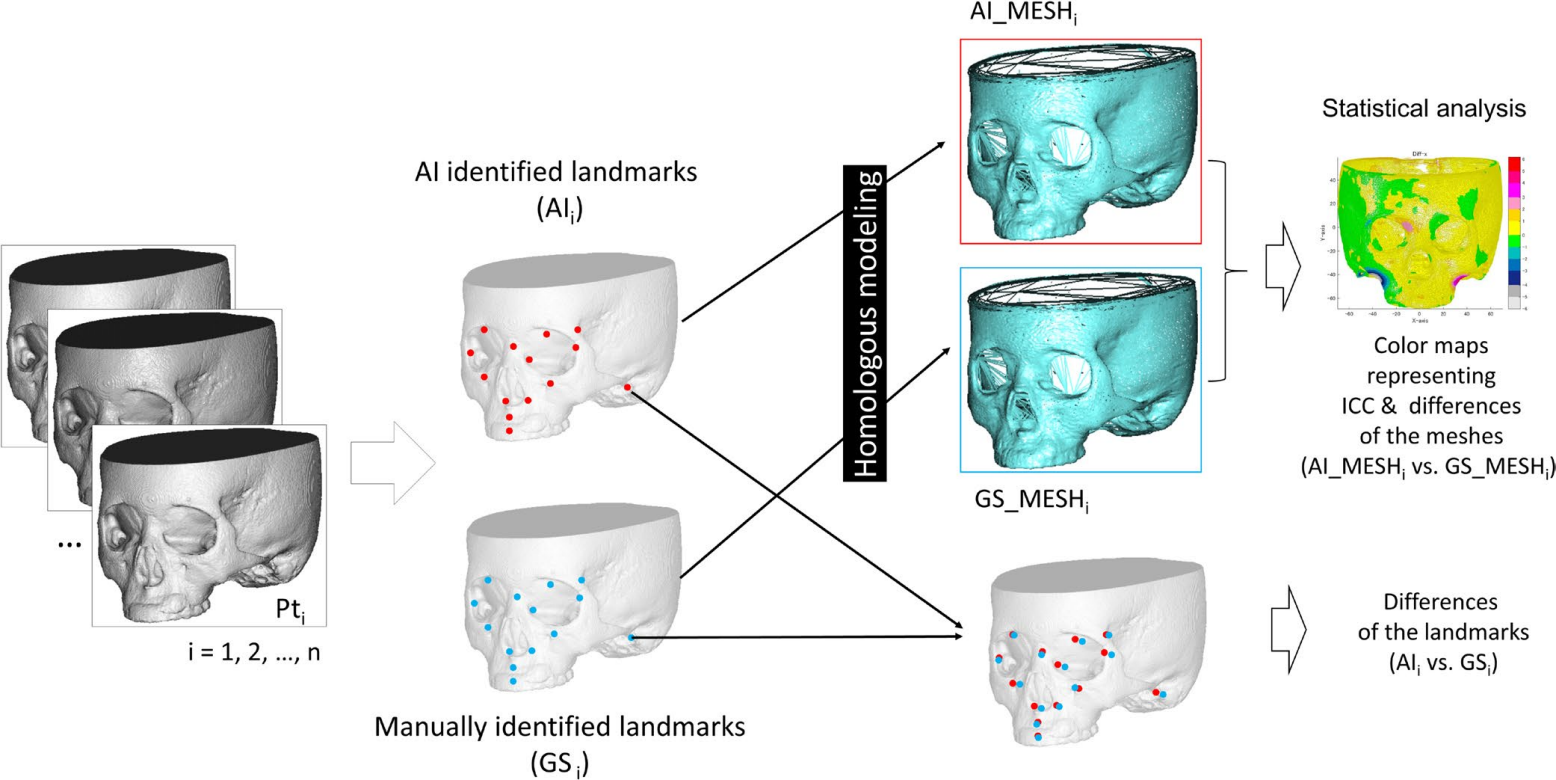
Accuracy measurements for the meshes based on the automatically identified landmarks (top) and those landmarks (bottom).

To evaluate the accuracy of mesh fitting based on the AI‐identified landmarks, the following experiment was conducted (Figure 2).

First, statistical homologous modelling [5] was conducted based on the two types of landmarks (GS and AI) as landmark anchors, and two types of mesh (GS_MESH and AI_MESH) were obtained from each patient and each maxilla and mandible 3‐D model. Homologous modelling is a high‐resolution template mesh fitting method that generates point clouds consisting of 54,426 and 16,438 data points for the crania and mandible (i.e., fitted mesh or semi‐landmark nodes). This technique permits the extraction of relevant surface anatomy from facial data while removing and/or smoothing out non‐relevant data, yielding high‐resolution 3D surface data that provide sufficient detail to facilitate quantitative assessment while maintaining small file sizes that are easily manipulated and portable to a range of visualisation technologies [4, 27]. A commercial software program implementing homologous modelling for this process (HBM‐Rugle, Medic Engineering Co., Kyoto, Japan) was used for the above process.

Second, the accuracy of AI_MESH when GS_MESH was set as the gold standard was calculated as the absolute difference (Supporting Information S4). Further, the intraclass correlation coefficient (ICC) estimates were calculated using a statistical software program (MATLAB R2022a; MathWorks, Natick, MA, USA) based on a single rater, absolute‐agreement, two‐way random‐effects model with ICC (1, 2) [28]. ‘Moderate’ and ‘substantial’ agreement were defined as ICC 0.40 to ≤ 0.60 and ICC 0.60 to ≤ 0.80, respectively, while ICC ≥ 0.81 indicated ‘almost perfect’ agreement [24]. The mean ICC was also calculated for the zygomatic bone, frontal bone and orbit, mastoid process, maxillary bone, mandibular condyle, coronoid process, mandibular ramus, mandibular angle, mandibular body and chin.

## 3 Results

### 3.1 AI System

Two systems have been developed to identify landmarks for the cranium and mandible. The identification time was within 2 s for both the cranium and mandible.

### 3.2 Errors in Identifying Landmarks

The mean error for landmark identification in the maxilla was 3.07 mm, whereas that in the mandible was 2.15 mm. The details are listed in Table 1 and the confidence ellipses are shown in Figure S4. The mean errors in the transverse, vertical, and anteroposterior directions were 1.13, 1.71, and 1.65 mm, respectively, in the maxilla; and 0.94, 1.21, and 1.04 mm in the mandible, respectively. Among the three directions, the vertical errors were the greatest.

**TABLE 1:** Absolute differences between the AI‐identified landmarks (AI) and the gold standard (GS) in the maxilla in the *X*‐, *Y*‐, and *Z*‐ axes (mm). SD, standard deviation; R, right; L, left.

Landmark			X		Y		Z		3D	
			Mean	SD	Mean	SD	Mean	SD	Mean	SD
1	Nasion		0.82	0.74	3.28	2.38	0.93	0.96	3.74	2.31
2	Anterior Nasal Spine		0.59	0.40	1.24	1.28	1.37	1.36	2.13	1.69
3	Prosthion		0.85	0.69	1.53	1.19	1.15	1.28	2.37	1.51
4	Frontozygomatic	R	1.65	1.60	1.66	1.49	1.66	1.62	3.28	2.20
5	Frontozygomatic	L	1.73	1.26	1.78	1.29	1.61	1.52	3.28	1.86
6	Apertion	R	0.87	0.64	2.17	1.83	0.88	0.64	2.76	1.65
7	Apertion	L	0.82	0.64	2.25	2.15	0.86	0.66	2.85	1.93
8	Jugale	R	1.05	1.22	1.66	1.46	2.50	2.27	3.51	2.55
9	Jugale	L	0.83	0.76	1.55	1.24	2.03	2.11	3.08	2.05
10	Orbital foramen	R	1.50	1.29	1.43	1.16	1.34	0.96	2.76	1.54
11	Orbital foramen	L	1.54	1.12	1.65	1.37	1.40	0.91	2.98	1.44
12	A‐Point		0.96	0.72	1.99	1.40	0.77	0.60	2.55	1.33
13	Incisive foramen		0.87	0.72	1.57	1.29	1.52	1.19	2.70	1.33
14	Posterior Nasal Spine		0.72	0.48	1.49	1.90	1.36	1.32	2.47	2.01
15	Basion		1.42	1.31	1.79	1.49	2.32	2.38	3.77	2.41
16	Foramen magnum		1.57	1.25	1.29	1.24	3.79	3.40	4.88	3.04
17	Mastoidale	R	1.55	1.34	1.06	0.87	1.93	1.39	2.96	1.72
18	Mastoidale	L	1.25	1.34	1.23	1.28	2.25	1.87	3.17	2.24
19	Rhinion		0.92	0.85	1.96	1.78	1.59	1.69	3.07	2.11
	Average		1.13	0.97	1.71	1.48	1.65	1.48	3.07	1.94
20	Mesial glenoid process	L	0.60	0.55	1.21	1.10	1.03	0.95	1.92	1.26
21	Mesial glenoid process	R	0.91	1.06	1.32	1.35	1.41	1.06	2.40	1.68
22	Lateral glenoid process	L	0.86	1.10	1.42	1.17	1.07	1.07	2.28	1.54
23	Lateral glenoid process	R	0.61	0.71	1.17	0.95	1.19	1.36	2.13	1.35
24	Coronoid process	L	0.92	0.92	1.08	1.16	1.00	0.91	2.00	1.41
25	Coronoid process	R	0.79	0.59	1.19	1.14	1.07	0.84	2.07	1.11
26	Sigmoid notch	L	0.64	0.63	0.68	0.74	1.12	0.75	1.63	0.97
27	Sigmoid notch	R	0.66	0.61	0.73	0.75	1.34	0.94	1.91	0.96
28	Mental foramen	L	1.16	1.05	1.09	1.18	1.10	0.72	2.11	1.52
29	Mental foramen	R	0.85	0.59	1.18	0.78	0.86	0.67	1.86	0.90
30	Pogonion		0.98	0.79	1.05	1.05	0.32	0.42	1.68	1.10
31	Menton		1.06	0.85	0.24	0.22	1.21	1.13	1.85	1.11
32	Gonion	L	0.85	0.83	1.25	0.91	0.89	0.73	1.93	1.18
33	Gonion	R	0.80	0.68	1.00	0.76	0.74	0.69	1.63	1.00
34	B‐Point		1.00	0.88	1.12	1.02	0.23	0.21	1.66	1.17
35	Antegonial notch	L	0.93	0.63	0.83	0.67	1.95	1.49	2.52	1.43
36	Antegonial notch	R	1.14	1.01	0.81	0.71	1.51	1.19	2.26	1.43
37	Lateral mandibular foramen	L	0.82	0.96	1.43	1.12	1.03	0.89	2.23	1.32
38	Lateral mandibular foramen	R	0.71	0.65	1.57	1.28	0.75	0.91	2.05	1.48
39	Infradentale		0.89	0.89	0.95	0.92	0.60	0.66	1.65	1.18
40	Postero‐superior condyle	L	1.12	1.10	1.60	1.16	0.49	0.70	2.27	1.39
41	Postero‐superior condyle	R	1.15	0.91	1.46	0.91	0.43	0.45	2.07	1.09
42	L6	L	0.75	0.93	1.14	1.07	1.51	1.61	2.31	1.85
43	L6	R	0.78	0.71	1.16	0.83	1.62	1.35	2.32	1.48
44	L3	L	0.78	0.67	0.72	0.96	0.70	0.60	1.44	1.12
45	L3	R	1.06	0.98	0.88	0.79	0.92	0.84	1.90	1.19
46	L7	L	0.84	0.69	1.17	1.23	2.12	2.09	2.84	2.19
47	L7	R	0.81	0.67	1.33	1.05	1.82	1.51	2.61	1.64
48	L1		0.94	1.09	0.90	0.85	0.97	0.90	1.85	1.37
49	Mandibular ramus	L	0.75	0.86	0.91	0.87	1.84	1.44	2.35	1.67
50	Mandibular ramus	R	1.03	0.62	0.95	0.68	1.21	1.07	2.01	1.17
51	Superior condyle	L	1.02	0.88	0.34	0.55	1.08	0.87	1.70	1.11
52	Superior condyle	R	1.09	0.83	0.44	0.51	1.21	0.83	1.89	0.94
53	Anterior condyle	L	1.56	1.45	1.40	1.13	0.67	0.74	2.43	1.68
54	Anterior condyle	R	1.57	1.38	1.92	1.37	0.74	0.68	2.84	1.68
55	Anterior mandibular ramus	L	0.94	0.84	1.49	1.32	0.79	0.69	2.17	1.38
56	Anterior mandibular ramus	R	1.02	0.89	2.14	1.68	0.98	0.85	2.85	1.66
57	Postero‐inferior mandibular ramus	L	0.86	0.86	1.44	1.40	0.38	0.48	1.95	1.44
58	Postero‐inferior mandibular ramus	R	1.05	0.92	1.73	1.62	0.63	0.60	2.35	1.66
59	Anterior mandibular foramen	L	1.22	1.06	1.55	1.38	0.95	0.77	2.47	1.50
60	Anterior mandibular foramen	R	1.07	0.82	1.62	1.51	0.83	0.72	2.39	1.49
61	Posterior mandibular foramen	L	0.66	0.77	1.44	1.27	1.18	1.00	2.20	1.49
62	Posterior mandibular foramen	R	0.68	0.60	1.67	1.38	1.22	1.29	2.42	1.67
63	Mesial mandibular foramen	L	1.07	0.76	1.78	1.57	1.16	1.29	2.67	1.79
64	Mesial mandibular foramen	R	1.20	0.88	1.81	1.21	1.02	1.07	2.61	1.51
	Average		0.94	0.85	1.21	1.05	1.04	0.93	2.15	1.38

### 3.3 Errors in Wire Mesh Fitting

**FIGURE 3:**
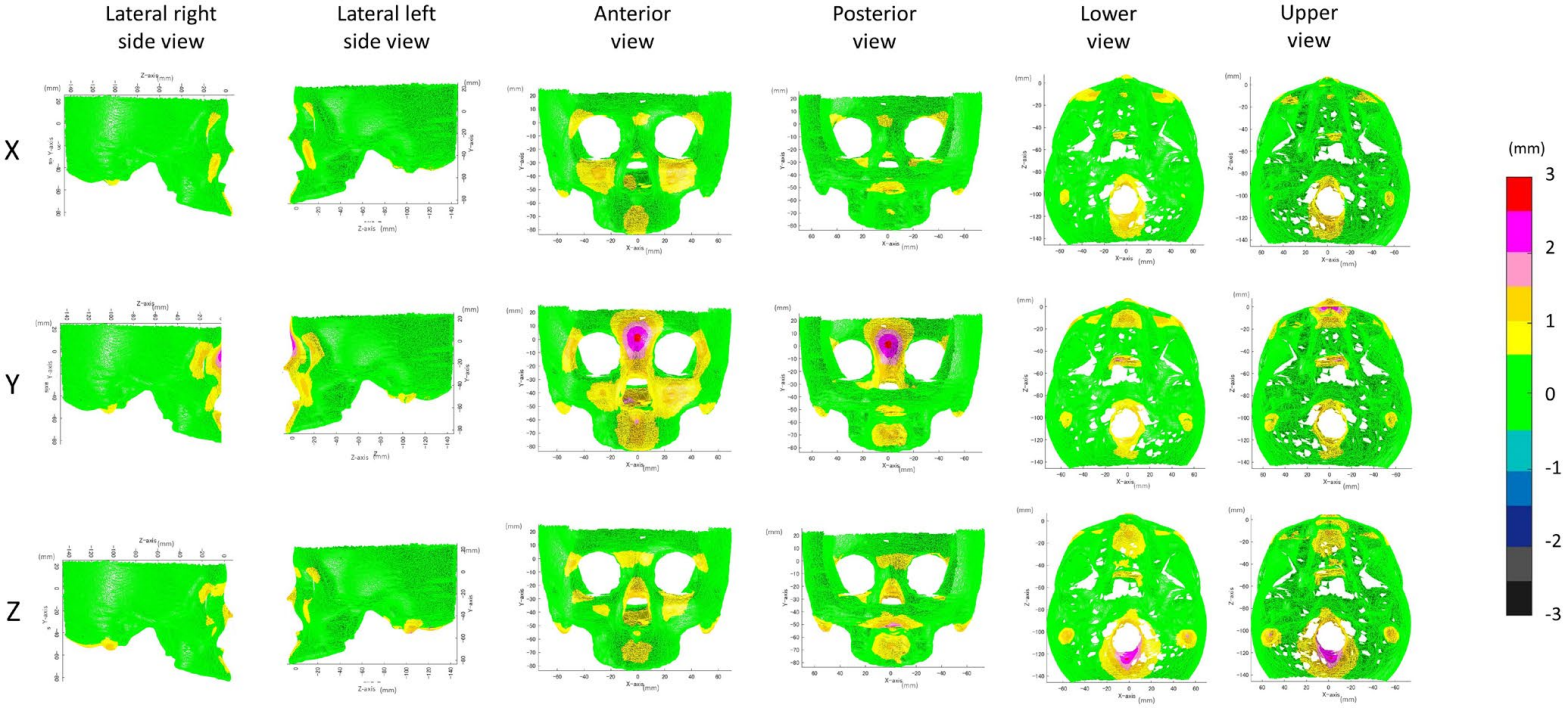
Absolute differences between mesh fitting based on the AI‐identified landmarks (AI_MESH) and the gold standard (GS_MESH) in the maxilla. X, transverse direction; Y, vertical direction, Z, antero‐posterior direction.

**FIGURE 4:**
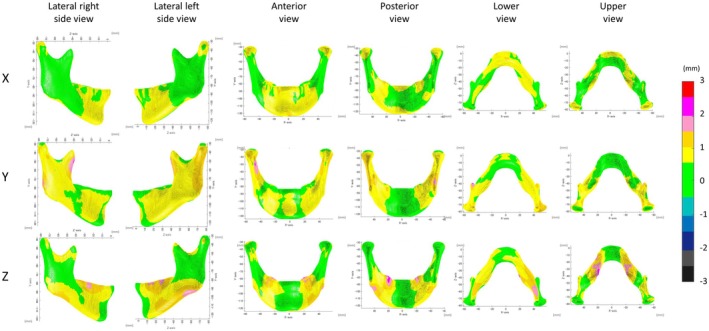
Absolute differences between mesh fitted based on the AI‐identified landmarks (AI_MESH) and the gold standard (GS_MESH) in the mandible. X, transverse direction; Y, vertical direction, Z, antero‐posterior direction.

The mean error for wire mesh fittings with AI‐identified landmarks for the maxilla and the mandible was 0.80 and 1.45 mm, respectively (Figures 3 and 4, Table 2). Errors in wire mesh fitting also showed the greatest errors in the vertical direction among the three directions; however, the errors in wire mesh fitting were smaller than those in landmark identification.

**TABLE 2:** Absolute differences of the point clouds between mesh fitting based on the AI‐identified landmarks (AI_MESH) and the gold standard (GS_MESH) in the *X*‐, *Y*‐, and *Z*‐axes.

Difference (mm)	X		Y		Z		3D	
	Mean	SD	Mean	SD	Mean	SD	Mean	SD
Maxilla								
Zygomatic bone	0.12	0.10	0.18	0.14	0.18	0.11	0.32	0.21
Frontal bone and orbit	0.35	0.24	0.68	0.57	0.35	0.25	0.94	0.64
Mastoid process	0.49	0.17	0.61	0.20	0.87	0.30	1.31	0.36
Maxillary bone	0.26	0.20	0.42	0.35	0.40	0.25	0.72	0.48
Total	0.31	0.26	0.49	0.43	0.38	0.29	0.80	0.57
Mandible								
Condyle	0.54	0.23	0.79	0.17	0.90	0.42	1.44	0.44
Coronoid	0.52	0.26	0.81	0.18	0.90	0.42	1.45	0.47
Ramus	0.55	0.24	0.83	0.17	0.84	0.34	1.43	0.34
Gonial angle	0.52	0.25	0.91	0.27	0.73	0.43	1.46	0.35
Body	0.46	0.24	0.98	0.23	0.62	0.41	1.42	0.30
Chin	0.40	0.19	1.12	0.26	0.62	0.34	1.51	0.25
Total	0.50	0.24	0.91	0.25	0.76	0.39	1.45	0.34

In the vertical direction (*y*‐axis), the greatest difference was observed in the chin (mean, 1.12 mm), followed by the body (mean, 0.98 mm). In the anteroposterior direction (*Z*‐axis), the ramus, coronoid region, and mastoid process showed the greatest differences (mean, 0.87–0.90 mm). In the transverse direction (*X*‐axis), The ramus showed the greatest difference in the transverse direction (*X*‐axis; mean, 0.55 mm).

According to the ICC, the agreement was almost perfect in all regions, indicating that it is acceptable for clinical application (Table 3; Figures S5‐1 and S5‐2).

**TABLE 3:** Intraclass correlation coefficient (ICC) between the point clouds between mesh fitting based on the AI‐identified landmarks (AI_MESH) and the gold standard (GS_MESH) in the *X*‐, *Y*‐, and *Z*‐axes.

ICC	X		Y		Z		3D	
	Mean	SD	Mean	SD	Mean	SD	Mean	SD
Maxilla								
Zygomatic bone	1.00	0.01	1.00	0.00	1.00	0.01	1.00	0.01
Frontal bone and orbit	0.98	0.05	0.99	0.02	0.98	0.04	0.94	0.11
Mastoid process	0.99	0.01	1.00	0.01	0.98	0.01	0.98	0.01
Maxillary bone	0.99	0.04	1.00	0.00	0.98	0.02	1.00	0.00
Total	0.97	0.07	1.00	0.01	0.98	0.03	0.98	0.06
Mandible								
Condyle	0.98	0.02	0.99	0.01	0.99	0.01	0.99	0.01
Coronoid	0.98	0.02	0.99	0.01	0.99	0.01	0.99	0.01
Ramus	0.98	0.02	0.99	0.01	0.99	0.01	0.99	0.00
Gonial angle	0.98	0.02	0.99	0.02	1.00	0.01	0.99	0.00
Body	0.99	0.01	0.99	0.02	1.00	0.01	0.99	0.00
Chin	0.99	0.01	0.98	0.02	1.00	0.00	0.99	0.01
Total	0.98	0.02	0.99	0.01	1.00	0.01	0.99	0.01

## 4 Discussion

This study was conducted in two phases. In the first phase, AI algorithms automatically identified landmarks. In the second phase, these landmarks were utilised for mesh fitting, an essential step in 3D surface cephalometry. This 3D surface cephalometry, introduced in our previous study [5], enables a comprehensive analysis of the curved surfaces of craniofacial structures, offering advantages over the linear and angular measurements of traditional cephalometry. The present study aimed to assess the accuracy of mesh fitting using AI‐identified landmarks to advance the clinical application of 3D surface analyses.

As a result of the first phase, the mean landmark errors were 3.07 mm in the maxilla and 2.15 mm in the mandible, which are relatively large but comparable to those reported in previous 3D studies [7, 8, 9, 11, 13, 15, 29]. However, the mesh fittings (second phase) based on these landmarks showed almost perfect agreement, which was accompanied by the errors of 0.80 mm in the maxilla and 1.45 mm in the mandible when compared to the gold standard. The average error of the proposed mesh fitting using the AI‐identified landmarks was < 2 mm and showed almost complete agreement in all regions, indicating that this approach is acceptable for orthodontists. The reason why the errors in the mesh fittings were relatively smaller than the landmarks is considered to be due to the mesh fitting algorithm. The algorithm used for mesh fitting was conducted by referring to the shape of the crania or mandibles, and the identified landmarks were used as anchors, which may reduce errors. In other words, our method is limited in terms of landmark identification. To enhance its accuracy, the present study introduced a second mesh fitting process, which helped correct the relatively large errors.

The mesh demonstrated acceptable accuracy; however, further refinements are needed in the initial step of landmark identification. Since no studies have evaluated mesh fitting using AI‐identified landmarks, the results of this study were compared with those of previous research on 3D landmark identification. To date, three methods have been primarily employed to identify 3D landmarks: knowledge‐based, template‐based, and learning‐based methods [10, 30]. Knowledge‐based methods involve applying mathematical calculations, such as the peak or lowest point, to 3D images, which are considered suitable for the normal group but not for robust identification in patients with deformed shapes [10]. A previous study using this method showed that the overall mean identification error was 2.01 mm with a standard deviation of 1.23 mm for all 20 landmarks in a 30 CBCT dataset [29]. Template‐based (or atlas‐based) methods fit a reference template image to a test image, and the manually identified landmarks in the template image are then transferred to the test image. A study using a template‐based method with statistical shape models after the projection of 3D CT data onto 2D spaces identified landmarks with a 3.64‐mm mean error [8]. Another template‐based study using an elastic transformation method after automatic segmentation of the 3D surfaces showed a 1.99‐mm mean error for 19 landmarks in an 18 CBCT data [7]. Finally, learning‐based methods use machine learning, including deep learning, which require relatively large training samples. A study segmented the mandible automatically and identified 9 landmarks using deep learning with an average error of less than 3 pixels (1 pixel = 0.76 × 0.76–1.27 × 1.27 mm) [9]. Recently, a study also showed an average error of 1.96 ± 0.78 mm for 16 landmarks using multi‐staged deep reinforcement learning [13]. Regardless of the method applied, several of these studies successfully showed an accuracy of < 2 mm (error of < 2 mm has been defined as ‘successful’ [7, 8, 9, 10, 13, 29, 30]). These studies, however, relied on a relatively small set of cephalometric points (fewer than 20 landmarks). Another learning‐based study with a relatively large number of landmarks showed a 3D point‐to‐point error of 3.63 mm for 93 landmarks using CT data, indicating difficulties in 3D landmarking with deep learning, owing to the small number of images included in the training samples [11]. These previous studies suggested that using any of the above three methods to identify 3D landmarks still showed greater errors, or most of the algorithms were evaluated based on a small set of cephalometric points [10]. A more recent study [15] using voxel grids of images at different spatial resolutions reported an error of 1.54 ± 0.87 mm for 32 landmarks, based on 143 CBCT datasets used for training and testing landmark detection. This suggests that the voxel grid method is promising for 3D landmark identification. However, because our ultimate goal was 3D surface cephalometry, which requires mesh fitting with surface‐identified landmarks, this study employed surface‐based recognition methodology. One notable limitation of this surface‐based approach is its reduced accuracy in detecting internal landmarks, such as the Sella. Although the voxel grid method shows potential for improving accuracy, further studies are needed to validate its efficacy. It is also noteworthy that a previous voxel‐based study [15] assessed only 32 out of 119 clinically relevant landmarks, with 18 focused on tooth positions. Consequently, the clinical accuracy of evaluating craniofacial morphology remains undetermined. At present, no system exists that can fully analyse craniofacial surface morphology through a combination of automated 3D landmark identification and mesh fitting. This study is the first to develop an AI‐supported, automatic, three‐dimensional surface cephalometry method.

The present study has six main limitations. First, the present study utilised CBCT images from a single vendor machine. These results may differ from those obtained using other machines. Second, the present study used samples showing skeletal Class 3 tendencies (mean ANB, 0.23°). Considering the basic principle of minimising unnecessary radiation exposure, it was impossible to obtain CBCT images from subjects with normal occlusions. Third, the study sample had limited ethnic diversity as it consisted primarily of Japanese participants. Fourth, the present study used manual landmarking, which was carried out by a trained operator as the gold standard, and this is still considered the only reliable method to evaluate the identified landmarks [31, 32]. Fifth, while the present study showed acceptable results in mesh fitting with AI‐identified landmarks, caution is warranted, as the automatic landmarking process itself still requires improvement, which will involve additional refinement. From the ethical and legal viewpoint that there remains a professional responsibility for the profession and patients, the clinical applicability of the AI system still requires final approval by expert orthodontists. Finally, in the present study, 3D reconstruction was performed manually, requiring approximately 30–60 min per patient. Given that an AI‐based system for 3D reconstruction has been previously reported [33, 34], which takes approximately 5 min, our future system aims to integrate both AI approaches to achieve fully automated 3D surface cephalometry. These AI systems in orthodontics have the potential to improve clinical workflow and productivity and thus enhance the clinical and research capabilities of surface‐based 3D cephalometry systems in the near future. Although traditional 2D cephalometry cannot always be fully replaced by 3D surface cephalometry, which involves relatively higher radiation, our previous research [5] has demonstrated that 3D surface cephalometry is valuable for patients with syndromic conditions affecting skeletal morphology. Specific cases where 3D cephalograms are essential will be identified in future studies.

## 5 Conclusion

Our surface‐based automatic recognition method yields relatively large errors. However, mesh fitting using AI‐identified landmarks as anchors enhanced their accuracy and showed almost complete agreement in all regions, indicating that this approach is acceptable when orthodontists can use a surface‐based 3D cephalometry system to evaluate craniomandibular morphology in clinical settings.

## Conflicts of Interest

The authors declare no conflicts of interest.

## Supporting information


**Figure S1.** Landmarks employed to examine the reliability of landmark identification. The landmarks selected after reliability examinations were designated as ‘included’ in parentheses in the caption. For further details, refer to Supporting Information S2.

**Figure S2.** The inter‐ and intra‐examiner reliability in the x‐ (top), y‐ (middle), and z‐ (bottom) axes for the cranial surface. (A) (blue bar) indicates intra‐examiner reliability for Examiner A; (B) (red bar), intra‐examiner reliability for Examiner B; (A, B) (green bar), inter‐examiner reliability. (a) > 2 mm of intra‐examiner reliability for Examiner A. (b) > 2 mm of intra‐examiner reliability for Examiner B. (c) > 2 mm of inter‐examiner reliability. Landmarks were excluded if they satisfied even one of the criteria a–c.

**Figure S3.** The inter‐ and intra‐examiner reliability in the x‐ (top), y‐ (middle), and z‐ (bottom) axes for the mandibular surface. (A) (blue bar) indicates intra‐examiner reliability for Examiner A; (B) (red bar), intra‐examiner reliability for Examiner B; (A, B) (green bar), inter‐examiner reliability. (a) > 2 mm of intra‐examiner reliability for Examiner A. (b) > 2 mm of intra‐examiner reliability for Examiner B. (c) > 2 mm of inter‐examiner reliability. (d) The same name landmarks as the landmarks showing > 2 mm of intra‐examiner reliability on the opposite side were deleted because we intended to employ a symmetrical landmark assignment. (e.g., if the right anterior border of the ramus (ABR [#19]) showed greater errors, then we excluded the left ABR [#18]). The landmarks were excluded if they satisfied even one of the criteria a–d.

**Figure S4.** 95% confidence ellipse for the errors of the AI‐identified landmarks (AI) when compared to the gold standard (GS).

**Figure S5‐1.** Intraclass correlation (ICC) between mesh fitted based on the AI‐identified landmarks (AI_MESH) and the gold standard (GS_MESH) in the maxilla. X, transverse direction; Y, vertical direction, Z, antero‐posterior direction.

**Figure S5‐2.** Intraclass correlation (ICC) between mesh fitted based on the AI‐identified landmarks (AI_MESH) and the gold standard (GS_MESH) in the mandible. X, transverse direction; Y, vertical direction, Z, antero‐posterior direction.

Data S1.

Data S2.

## Data Availability

The data that support the findings of this study are available on request from the corresponding author. The data are not publicly available due to privacy or ethical restrictions.
