# Supplementary figures and images for: Development of Artificial Intelligence‐Supported Automatic Three‐Dimensional Surface Cephalometry
Source: Orthod Craniofac Res. 2025 Mar 4;28(4):636–46. doi: 10.1111/ocr.12914 (PMC12233043; doi:10.1111/ocr.12914)

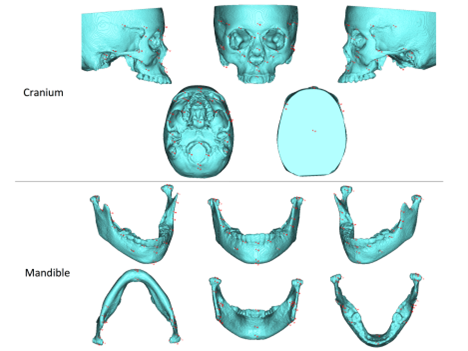

Supplement: Supplementary file 1 — Figure S1. Landmarks employed to examine the reliability of landmark identification. The landmarks selected after reliability examinations were designated as ‘included’ in parentheses in the caption. For further details, refer to Supporting Information S2. [file OCR-28-636-s006.tif]

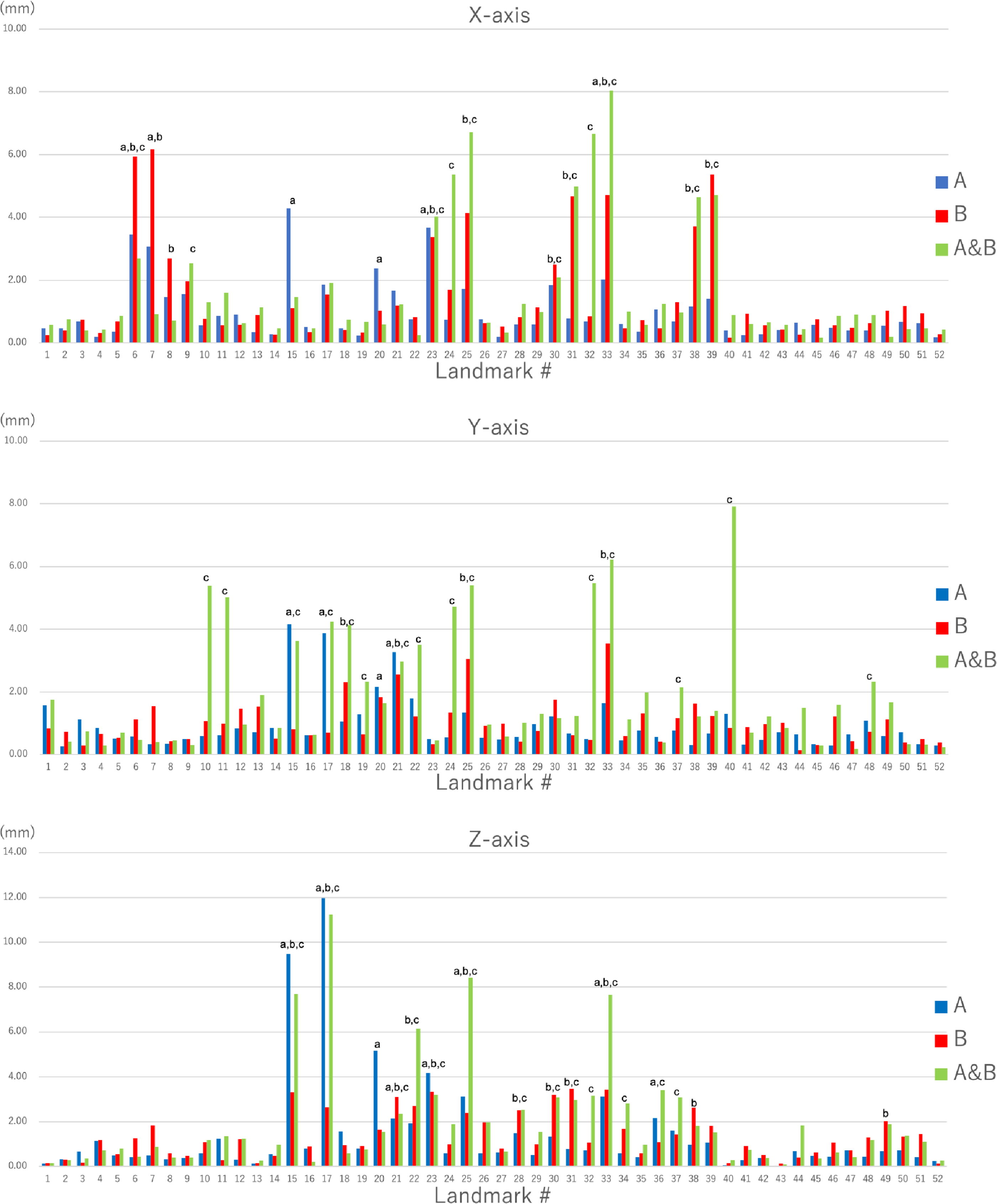

Supplement: Supplementary file 2 — Figure S2. The inter‐ and intra‐examiner reliability in the x‐ (top), y‐ (middle), and z‐ (bottom) axes for the cranial surface. (A) (blue bar) indicates intra‐examiner reliability for Examiner A; (B) (red bar), intra‐examiner reliability for Examiner B; (A, B) (green bar), inter‐examiner reliability. (a) > 2 mm of intra‐examiner reliability for Examiner A. (b) > 2 mm of intra‐examiner reliability for Examiner B. (c) > 2 mm of inter‐examiner reliability. Landmarks were excluded if they satisfied even one of the criteria a–c. [file OCR-28-636-s005.tif]

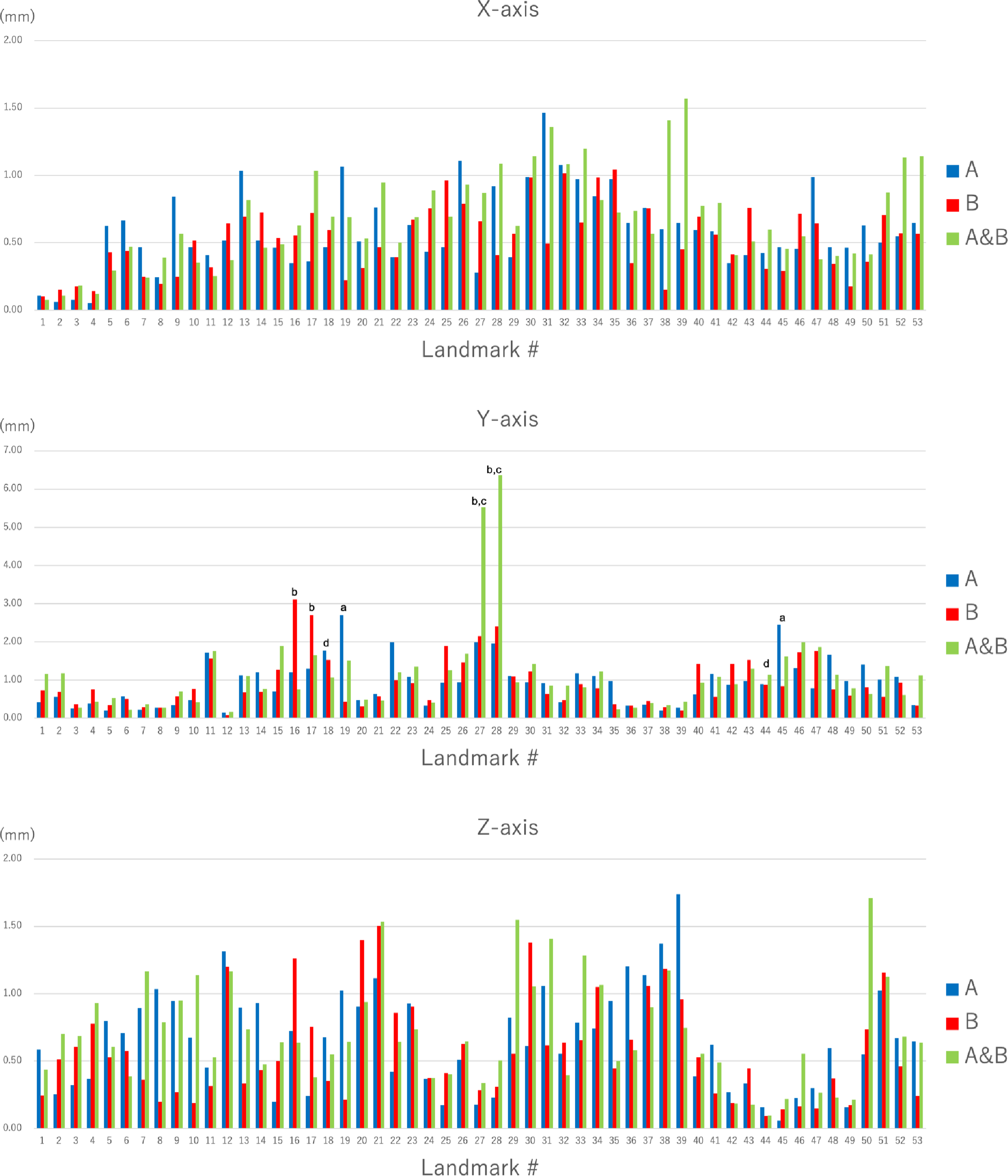

Supplement: Supplementary file 3 — Figure S3. The inter‐ and intra‐examiner reliability in the x‐ (top), y‐ (middle), and z‐ (bottom) axes for the mandibular surface. (A) (blue bar) indicates intra‐examiner reliability for Examiner A; (B) (red bar), intra‐examiner reliability for Examiner B; (A, B) (green bar), inter‐examiner reliability. (a) > 2 mm of intra‐examiner reliability for Examiner A. (b) > 2 mm of intra‐examiner reliability for Examiner B. (c) > 2 mm of inter‐examiner reliability. (d) The same name landmarks as the landmarks showing > 2 mm of intra‐examiner reliability on the opposite side were deleted because we intended to employ a symmetrical landmark assignment. (e.g., if the right anterior border of the ramus (ABR [#19]) showed greater errors, then we excluded the left ABR [#18]). The landmarks were excluded if they satisfied even one of the criteria a–d. [file OCR-28-636-s002.tif]

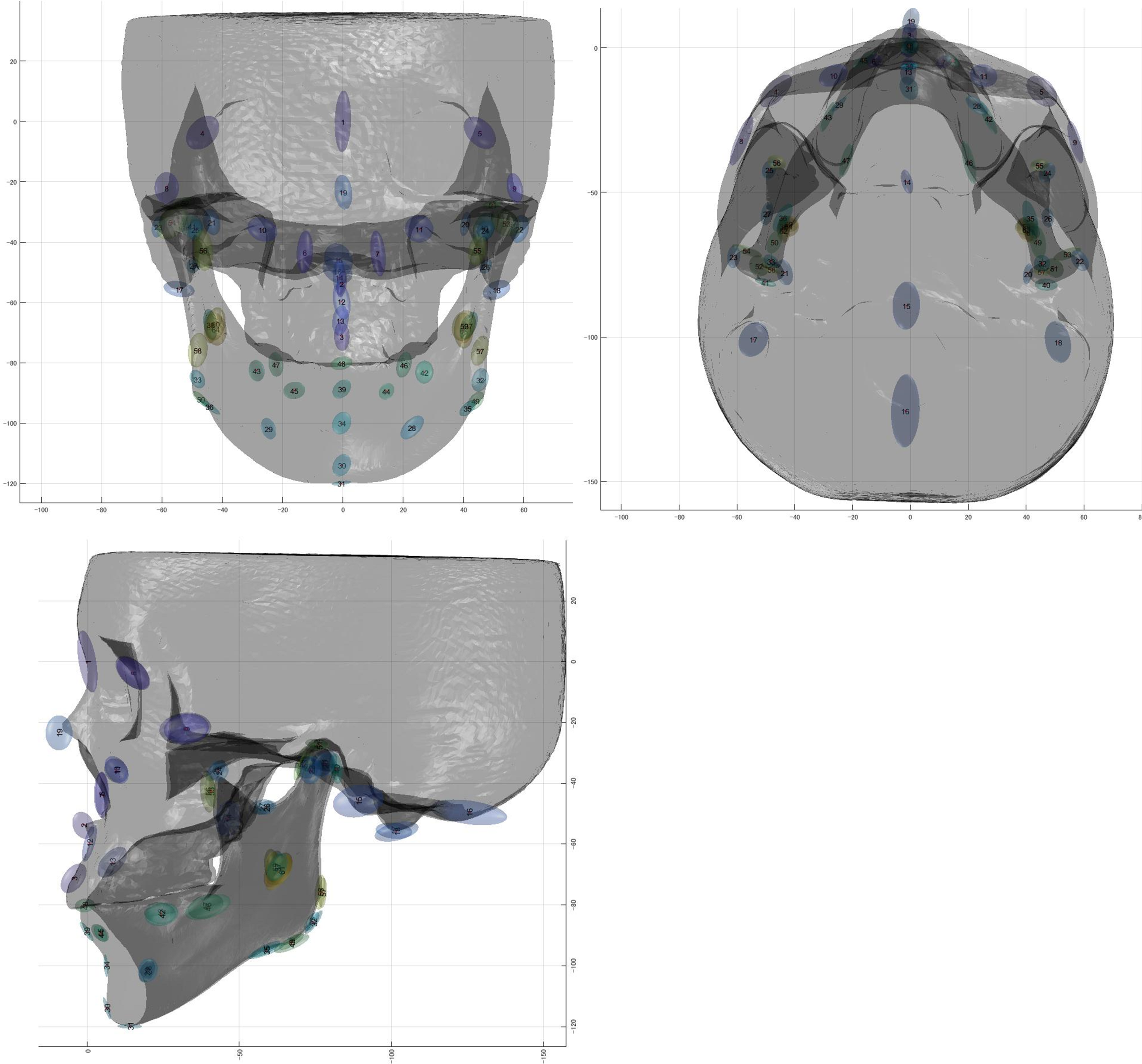

Supplement: Supplementary file 4 — Figure S4. 95% confidence ellipse for the errors of the AI‐identified landmarks (AI) when compared to the gold standard (GS). [file OCR-28-636-s008.tif]

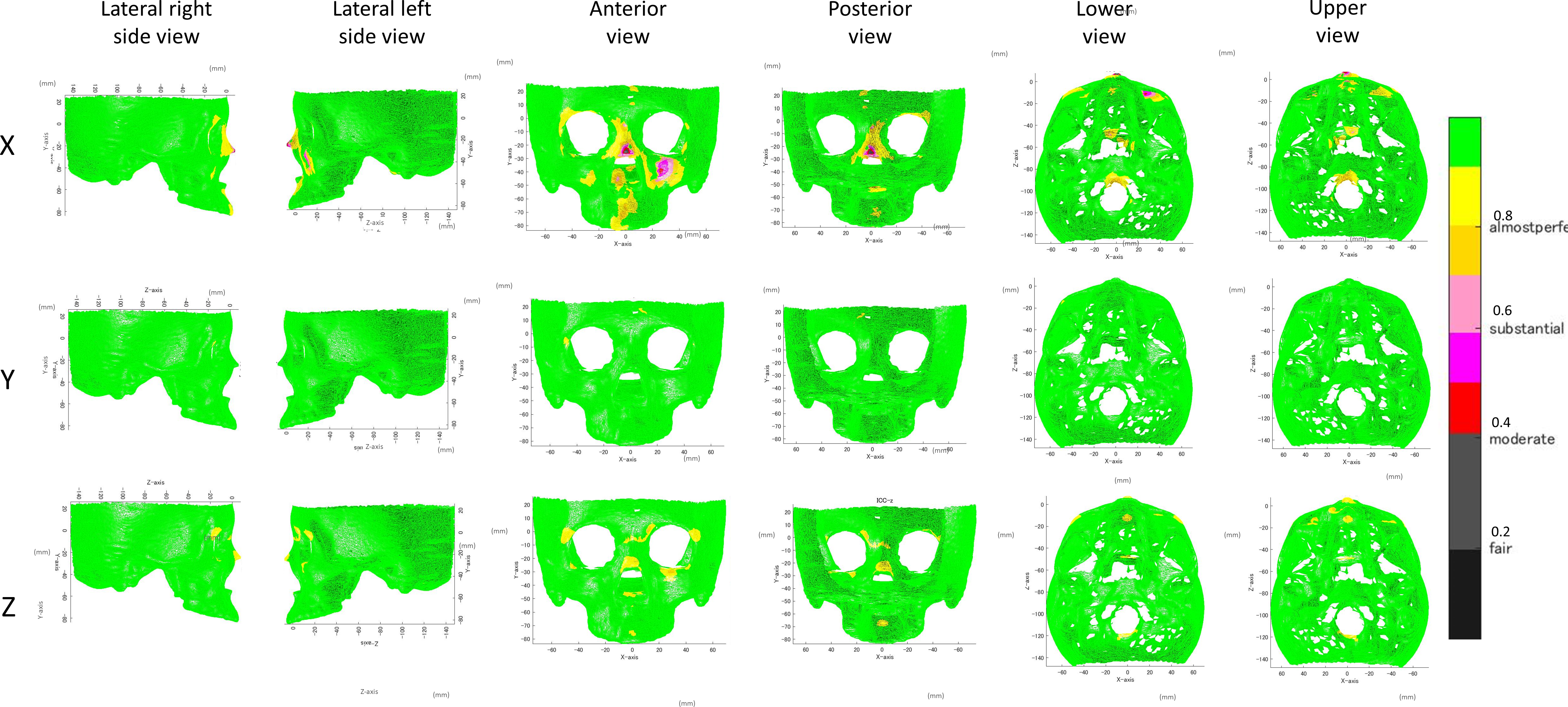

Supplement: Supplementary file 5 — Figure S5‐1. Intraclass correlation (ICC) between mesh fitted based on the AI‐identified landmarks (AI_MESH) and the gold standard (GS_MESH) in the maxilla. X, transverse direction; Y, vertical direction, Z, antero‐posterior direction. [file OCR-28-636-s004.tif]

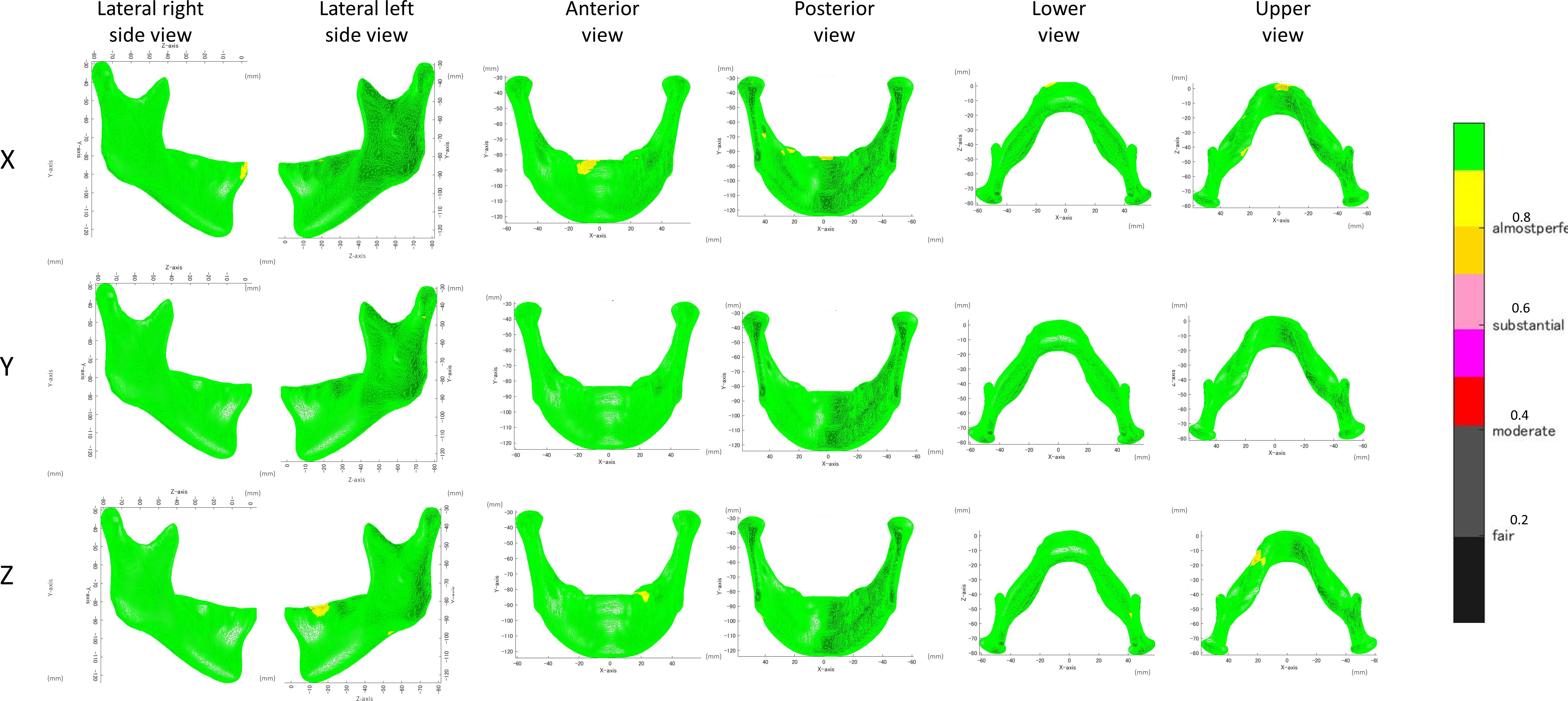

Supplement: Supplementary file 6 — Figure S5‐2. Intraclass correlation (ICC) between mesh fitted based on the AI‐identified landmarks (AI_MESH) and the gold standard (GS_MESH) in the mandible. X, transverse direction; Y, vertical direction, Z, antero‐posterior direction. [file OCR-28-636-s003.tif]
