# Supplementary material for: Development of Artificial Intelligence‐Supported Automatic Three‐Dimensional Surface Cephalometry
Source: Orthod Craniofac Res. 2025 Mar 4;28(4):636–46. doi: 10.1111/ocr.12914 (PMC12233043; doi:10.1111/ocr.12914)
Supplement: Supplementary file 7 — Data S1. [file OCR-28-636-s001.docx]

**Supplementary Text S1: Definition of the landmarks**

Definition of the cranial landmarks

1. The midpoint of the frontonasal suture (Nasion). ^1^
2. The most anterior midpoint of the anterior nasal spine of the maxilla (Anterior Nasal Spine). ^1^
3. The midpoint at the most anterior edge of the inferior alveolar ridge of the maxilla (Prosthion). ^2^
4. The most medial and anterior point of the right frontozygomatic suture at the lateral orbital rim (right Frontozygomatic). ^1^
5. The most medial and anterior point of the left frontozygomatic suture at the lateral orbital rim (left Frontozygomatic). ^1^
6. The most lateral point of the piriform aperture (right Apertion). ^2^
7. The most lateral point of the piriform aperture (left Apertion). ^2^
8. The most concave point between the lateral margin of the upper zygomatic bone and the upper margin of the zygomatic arch (right Jugale). ^3^
9. The most concave point between the lateral margin of the upper zygomatic bone and the upper margin of the zygomatic arch (left Jugale). ^3^
10. The most superior point of the infraorbital foramen (right Orbital foramen).*
11. The most superior point of the infraorbital foramen (left Orbital foramen).*
12. The point of maximum concavity in the midline of the alveolar process of the maxilla (A-Point). ^1^
13. The most posterior point of the incisive foramen (Incisive foramen).*
14. The most posterior midpoint of the posterior nasal spine of the palatine bone (Posterior Nasal Spine). ^1^
15. The most anterior point of the foramen magnum (Basion). ^1^
16. The most posterior point of the foramen magnum (Foramen magnum).*
17. The most inferior point of the mastoid process (right Mastoidale). ^2^
18. The most inferior point of the mastoid process (left Mastoidale). ^2^
19. The midline point at the inferior free end of the internasal suture (Rhinion). ^2^

Definition of the mandibular landmarks

1. The most medial point of the glenoid process of the mandible (left mesial glenoid process).*
2. The most medial point of the glenoid process of the mandible (right mesial glenoid process).*
3. The most lateral point of the glenoid process of the mandible (left lateral glenoid process).^2^
4. The most lateral point of the glenoid process of the mandible (right lateral glenoid process). ^2^
5. The most superior point on the coronoid process of the mandibular ramus (left coronoid process). ^1^
6. The most superior point on the coronoid process of the mandibular ramus (right coronoid process). ^1^
7. The point of maximum concavity on the superior border of the sigmoid notch (left sigmoid notch).*
8. The point of maximum concavity on the superior border of the sigmoid notch (right sigmoid notch).*
9. The most anterior point of the border of the mental foramen (left mental foramen). ^2^
10. The most anterior point of the border of the mental foramen (right mental foramen). ^2^
11. The most anterior midpoint of the chin on the outline of the mandibular symphysis (Pogonion).^1^
12. The most inferior midpoint of the chin on the outline of the mandibular symphysis (Menton). ^1^
13. The point at each mandibular angle that is defined by dropping a perpendicular line from the intersection point of the tangent lines to the posterior margin of the mandibular vertical ramus and inferior margin of the mandibular body or horizontal ramus (left Gonion). ^1^
14. The point at each mandibular angle that is defined by dropping a perpendicular from the intersection point of the tangent lines to the posterior margin of the mandibular vertical ramus and inferior margin of the mandibular body or horizontal ramus (right Gonion). ^1^
15. The point of maximum concavity in the midline of the alveolar process of the mandible (B-Point). ^1^
16. The deepest point of the antegonial notch (left antegonial notch). ^4^
17. The deepest point of the antegonial notch (right antegonial notch). ^4^
18. The most lateral point of the border of the mandibular foramen (left lateral mandibular foramen).*
19. The most lateral point of the border of the mandibular foramen (right lateral mandibular foramen).*
20. The midline point at the most anterior edge on the superior alveolar ridge of the mandible (Infradentale). ^2^
21. The most postero-superior point on the mandibular condyle (left postero-superior condyle). ^5^
22. The most postero-superior point on the mandibular condyle (right postero-superior condyle). ^5^
23. The point on the inferior alveolar ridge inferior to the crown of the mandibular first molar (left L6). ^2^
24. The point on the inferior alveolar ridge inferior to the crown of the mandibular first molar (right L6). ^2^
25. The point on the inferior alveolar ridge inferior to the crown of the mandibular canine (left L3). ^2^
26. The point on the inferior alveolar ridge inferior to the crown of the mandibular canine (right L3). ^2^
27. The point on the inferior lingual alveolar ridge inferior to the crown of the mandibular second molar (left L7).*
28. The point on the inferior lingual alveolar ridge inferior to the crown of the mandibular second molar (right L7).*
29. The most superior midpoint between the lower central incisors on the lingual surface (L1).*
30. The contact point of the mandibular plane on the inferior margin of the mandibular ramus (left mandibular ramus).*
31. The contact point of the mandibular plane on the inferior margin of the mandibular ramus (right mandibular ramus).*
32. The most superior point of the mandibular condyle (left superior condyle).*
33. The most superior point of the mandibular condyle (right superior condyle).*
34. The most anterior point of the mandibular condyle (left anterior condyle).*
35. The most anterior point of the mandibular condyle (right anterior condyle).*
36. The most anterior point on the anterior border of the mandibular ramus (left anterior mandibular ramus). ^5^
37. The most anterior point on the anterior border of the mandibular ramus (right anterior mandibular ramus). ^5^
38. The contact point of the ramus plane on the postero-inferior margin of the mandibular ramus (left postero-inferior mandibular ramus).*
39. The contact point of the ramus plane on the postero-inferior margin of the mandibular ramus (right postero-inferior mandibular ramus).*
40. The most anterior point of the border of the mandibular foramen (left anterior mandibular foramen).*
41. The most anterior point of the border of the mandibular foramen (right anterior mandibular foramen).*
42. The most posterior point of the border of the mandibular foramen (left posterior mandibular foramen).*
43. The most posterior point of the border of the mandibular foramen (right posterior mandibular foramen).*
44. The most mesial point of the border of the mandibular foramen (left mesial mandibular foramen).*
45. The most mesial point of the border of the mandibular foramen (right mesial mandibular foramen).*

* indicates the landmarks that were defined in the previous study ^6^.

**Supplementary Text S2: Selection of landmarks**

Seven randomly selected CBCT data were employed to determine the inter-examiner reliability of landmark identification and to select landmarks used for mesh-fittings in our previous study ^6^. Each 3D image, scaled to 75% of its actual size, was displayed on a 17-in LCD monitor (1701FP, Dell Inc., Round Rock, TX, USA). The positions of 52 and 53 landmarks on the cranial and mandibular surfaces, respectively (Supplementary Figure 1), were identified by a visual inspection of the image and digitized using a computer mouse cursor and commercial software program (HBM Rugle, Medic Engineering Co., Kyoto, Japan). Landmark identification was repeated on two separate occasions by examiners A (orthodontist YU) and B, orthodontists with more than seven years of clinical experience each; there was an interval of one week between sessions. The absolute distances between sessions were calculated, and the landmarks satisfying the following criteria were excluded from further calculation steps:

Criterion 1: intra-examiner distance > 2 mm. ^7^

Criterion 2: right (left) counterpart landmarks satisfying Criterion 1 for the left (right) half.

Supplementary Figures 2 and 3 show the inter/intra-observer distances of the coordinates of the landmarks. Thirty-three (#6-#11, #15, #17-#41, #48, #49 in Supplementary Fig. 1) and 8 landmarks (#16-#19, #27, #28, #46, #47 in Supplementary Fig. 1) were excluded from mesh-fitting process for the cranial and mandibular surfaces, respectively. The mesh-fitting process therefore employed 19 and 45 landmarks for the cranial and mandibular surfaces, respectively.

**Supplementary Text S3: Network architecture and training**

Network architecture

As an input, 20,000 points (coordinates + normal vectors) on the polygonal surface were randomly sampled in a stereolithography file containing the CBCT data. The system architecture was composed of the following two parts. The first part of the network was PointNet++ ^6^, which consists of layers for set abstractions generating a 1,024 dimensional feature vector. The latter part of the network consists of three linear layers (with ReLU activations for the first two layers), the final output of which is a vector of dimension NUM_LM * 3, where NUM_LM is the number of landmark points. For regularization, a dropout of probability 0.3 is applied after the first linear layer and another dropout of probability 0.2 is applied after the second linear layer. Each of the cranium (NUM_LM = 19) and mandible (NUM_LM = 45) landmarks were trained with the respective training data.

Training

The mean square error between the predicted landmarks and the GS was used as the loss function for training of the network. To stabilize the network output against various translations of the input, the input points are first translated so that the center of gravity equals the origin (0, 0, 0). With the dataset stored for system training, the networks for the cranium and mandible were trained for 20,000 epochs each. During training, random scaling, shift (translation) and addition of random noise were applied to the input point coordinates for data augmentation. For system training for the cranium, 13 subjects were excluded due to poor image quality of the cranial CBCT.

**Supplementary Text S4: Calculation of the absolute differences**

The calculation of the absolute differences (Diff_x, Diff_y, Diff_z, Diff_3D) is defined as follows:

Diff_x = |xi - xi’|, where xi represents the x-coordinate of semilandmark i in the AI_ MESH, and xi’ represents the corresponding x-coordinate in the GS_ MESH.

Diff_y = |yi - yi’|, where yi represents the y-coordinate of semilandmark i in the AI_ MESH and yi’ represents the corresponding y-coordinate in the GS_MESH.

Diff_z = |zi - zi’|, where zi represents the z-coordinate of semilandmark i in the AI_MESH, and zi’ represents the corresponding z-coordinate in the GS_MESH.

Diff_3D = √[(xi - xi’)² + (yi - yi’)² + (zi - zi’)²], where xi, yi, and zi represent the coordinates of semilandmark i in the AI_MESH, and xi’, yi’, and zi’ represent the corresponding coordinates in the GS_MESH.

**References**

1. Swennen GRJ, Schutyser F, Hausamen J-E. Three-dimensional cephalometry: a color atlas and manual: *Springer Science & Business Media*; 2005.

2. Guyomarc'h P, Dutailly B, Charton J, et al. Anthropological facial approximation in three dimensions (AFA3D): Computer-assisted estimation of the facial morphology using geometric morphometrics. *Journal of Forensic Sciences* 2014;59(6):1502-16.

3. Kato Y, Kizu Y, Tonogi M, Ide Y, Yamane GY. Internal structure of zygomatic bone related to Zygomatic fixture. *Journal of Oral and Maxillofacial Surgery* 2005;63(9):1325-29.

4. Davidovitch M, Eleftheriadi I, Kostaki A, Shpack N. The use of Bjork's indications of growth for evaluation of extremes of skeletal morphology. *European Journal of Orthodontics* 2016;38(6):555-62.

5. Williams FL, Richtsmeier JT. Comparison of mandibular landmarks from computed tomography and 3D digitizer data. *Clinical Anatomy* 2003;16(6):494-500.

6. Tsukiboshi Y, Tanikawa C, Yamashiro T. Surface-based 3-dimensional cephalometry: An objective analysis of cranio-mandibular morphology. *Am J Orthod Dentofac*. 2020;158(4):535-546.

7. Forsyth DB, Davis DN. Assessment of an automated cephalometric analysis system. *Eur J Orthodont.* 1996;18(5):471-478.

8. Qi CR, Yi L, Su H, Guibas LJ. PointNet++: Deep hierarchical feature learning on point sets in a metric spac: *arXiv*; 2017.
