# Supplementary material for: Development of Artificial Intelligence‐Supported Automatic Three‐Dimensional Surface Cephalometry
Source: Orthod Craniofac Res. 2025 Mar 4;28(4):636–46. doi: 10.1111/ocr.12914 (PMC12233043; doi:10.1111/ocr.12914)
Supplement: Supplementary file 8 — Data S2. [file OCR-28-636-s007.docx]

**Figure S1.** Landmarks employed to examine the reliability of landmark identification. The landmarks selected after reliability examinations were designated as "included" in parentheses in the caption. For further details, refer to Supplementary Text 2.

Definition of the cranium landmarks

1. Nasion (included, Supplementary Text 1, landmark #1).

2. Anterior Nasal Spine (included, Supplementary Text 1, landmark #2).

3. Prosthion (included, Supplementary Text 1, landmark #3).

4. Right Frontozygomatic (included, Supplementary Text 1, landmark #4).

5. Left Frontozygomatic (included, Supplementary Text 1, landmark #5).

6. The most superior point of the right supraorbital rim (excluded).

7. The most superior point of the left supraorbital rim (excluded).

8. The most inferior point of the right infraorbital rim (right Orbitale, excluded).

9. The most inferior point of the left infraorbital rim (left Orbitale, excluded).

10. The most medial point of the right orbital rim (excluded).

11. The most medial point of the left orbital rim (excluded).

12. Right Apertion (included, Supplementary Text 1, landmark #6).

13. Left Apertion (included, Supplementary Text 1, landmark #7).

14. Right Jugale (included, Supplementary Text 1, landmark #8).

15. The most concave and lateral point of inferior border of the zygomatic process of the temporal bone at the position of the glenoid fossa (right, excluded).

16. Left Jugale (included, Supplementary Text 1, landmark #9).

17. The most concave and lateral point of inferior border of the zygomatic process of the temporal bone at the position of the glenoid fossa (left, excluded).

18. The most antero-medial point of the linea temporalis superior (right Frontotemporale, excluded).

19. The most antero-medial point of the linea temporalis superior (left Frontotemporale, excluded).

20. The junction of the sphenoid, temporal, parietal and frontal bones (right Pterion, excluded).

21. The junction of the sphenoid, temporal, parietal and frontal bones (left Pterion, excluded).

22. The most postero-inferior midline point of the external occipital protuberance (excluded).

23. The most superior point on the convexity of the calvarium from the Frankfurt plane (excluded).

24. The most supero-medial point on the maxillary inflexion between the zygomaxillare and the ectomolare (right Submaxillar curvature, excluded).

25. The most concave point between the lateral margin of the lower zygomatic bone and the lower margin of the zygomatic arch (right, excluded).

26. The most inferior point of the zygomatico-temporal suture (right Zygotemporale inferior, excluded).

27. The most posterior point of the right frontozygomatic suture (right Frontomalare temporale, excluded).

28. The most superior point of the zygomatico-temporal suture (right Zygotemporale superior, excluded).

29. The point of inflexion where the braincase curves laterally into the supraglenoid gutter, in coronal plane of the mandibular fossa (right, excluded).

30. The most superior point of the right external acoustic meatus (right Porion, excluded).

31. The most inferior point of the external acoustic meatus (left, excluded).

32. The most supero-medial point on the maxillary inflexion between the zygomaxillare and the ectomolare (left Submaxillar curvature, excluded).

33. The most concave point between the lateral margin of the lower zygomatic bone and the lower margin of the zygomatic arch (left, excluded).

34. The most inferior point of the zygomatico-temporal suture (left Zygotemporale inferior, excluded).

35. The most posterior point of the left frontozygomatic suture (left Frontomalare temporale, excluded).

36. The most superior point of the zygomatico-temporal suture (left Zygotemporale superior, excluded).

37. The point of inflexion where the braincase curves laterally into the supraglenoid gutter, in coronal plane of the mandibular fossa (left, excluded).

38. The most superior point of the left external acoustic meatus (left Porion, excluded).

39. The most inferior point of the external acoustic meatus (left, excluded).

40. The most anterior midline point on the frontal bone (Glabella, excluded).

41. Left Orbital foramen (included, Supplementary Text 1, landmark #10).

42. Left Orbital foramen (included, Supplementary Text 1, landmark #11).

43. A-Point (included, Supplementary Text 1, landmark #12).

44. Incisive foremen (included, Supplementary Text 1, landmark #13).

45. Posterior Nasal Spine (included, Supplementary Text 1, landmark #14).

46. Basion (included, Supplementary Text 1, landmark #15).

47. Foramen magnum (included, Supplementary Text 1, landmark #16).

48. The most lateral point of the foramen magnum (right, excluded).

49. The most lateral point of the foramen magnum (left, excluded).

50. Right Mastoidale (included, Supplementary Text 1, landmark #17).

51. Left Mastoidale (included, Supplementary Text 1, landmark #18).

52. Rhinion (included, Supplementary Text 1, landmark #19).

Definition of the mandibular landmarks

1 Left mesial glenoid process (included, Supplementary Text 1, landmark #20).

2 Right mesial glenoid process (included, Supplementary Text 1, landmark #21).

3 Left lateral glenoid process (included, Supplementary Text 1, landmark #22).

4 Right lateral glenoid process (included, Supplementary Text 1, landmark #23).

5 Left coronoid process (included, Supplementary Text 1, landmark #24)

6 Right coronoid process (included, Supplementary Text 1, landmark #25)

7 Left sigmoid notch (included, Supplementary Text 1, landmark #26)

8 Right sigmoid notch (included, Supplementary Text 1, landmark #27)

9 Left mental foramen (included, Supplementary Text 1, landmark #28)

10 Right mental foramen (included, Supplementary Text 1, landmark #29)

11 Pogonion (included, Supplementary Text 1, landmark #30)

12 Menton (included, Supplementary Text 1, landmark #31)

13 Left Gonion (included, Supplementary Text 1, landmark #32)

14 Right Gonion (included, Supplementary Text 1, landmark #33)

15 B-Point (included, Supplementary Text 1, landmark #34)

16 The midpoint of the superior mental spine (excluded).

17 The midpoint of the inferior mental spine (excluded).

18 The point where the anterior border of the ramus and occlusal plane intersect (left ABR, excluded).

19 The point where the anterior border of the ramus and occlusal plane intersect (right ABR, excluded).

20 Left antegonial notch (included, Supplementary Text 1, landmark #35)

21 Right antegonial notch (included, Supplementary Text 1, landmark #36)

22 Left lateral mandibular foramen (included, Supplementary Text 1, landmark #37)

23 Right lateral mandibular foramen (included, Supplementary Text 1, landmark #38)

24 Infradentale (included, Supplementary Text 1, landmark #39)

25 Left postero-superior condyle (included, Supplementary Text 1, landmark #40)

26 Right postero-superior condyle (included, Supplementary Text 1, landmark #41)

27 The point of maximum concavity on the posterior border of the mandibular ramus (left, excluded).

28 The point of maximum concavity on the posterior border of the mandibular ramus (right, excluded).

29 Left L6 (included, Supplementary Text 1, landmark #42)

30 Right L6 (included, Supplementary Text 1, landmark #43)

31 Left L3 (included, Supplementary Text 1, landmark #44)

32 Right L3 (included, Supplementary Text 1, landmark #45)

33 Left L7 (included, Supplementary Text 1, landmark #46)

34 Right L7 (included, Supplementary Text 1, landmark #47)

35 L1 (included, Supplementary Text 1, landmark #48)

36 Left mandibular ramus (included, Supplementary Text 1, landmark #49)

37 Right mandibular ramus (included, Supplementary Text 1, landmark #50)

38 Left superior condyle (included, Supplementary Text 1, landmark #51)

39 Right superior condyle (included, Supplementary Text 1, landmark #52)

40 Left anterior condyle (included, Supplementary Text 1, landmark #53)

41 Right anterior condyle (included, Supplementary Text 1, landmark #54)

42 Left anterior mandibular ramus (included, Supplementary Text 1, landmark #55)

43 Right anterior mandibular ramus (included, Supplementary Text 1, landmark #56)

44 Left postero-inferior mandibular ramus (included, Supplementary Text 1, landmark #57)

45 Right postero-inferior mandibular ramus (included, Supplementary Text 1, landmark #58)

46 The contact point of ramus plane on the postero-inferior margin of the mandibular ramus (left, excluded).

47 The contact point of ramus plane on the postero-inferior margin of the mandibular ramus (right, excluded).

48 Left anterior mandibular foramen (included, Supplementary Text 1, landmark #59)

49 Right anterior mandibular foramen (included, Supplementary Text 1, landmark #60)

50 Left posterior mandibular foramen (included, Supplementary Text 1, landmark #61)

51 Right posterior mandibular foramen (included, Supplementary Text 1, landmark #62)

52 Left mesial mandibular foramen (included, Supplementary Text 1, landmark #63)

53 Right mesial mandibular foramen (included, Supplementary Text 1, landmark #64)

b,c

**Figure S2.** The inter- and intra-examiner reliability in the x- (top), y- (middle), and z- (bottom) axes for the cranial surface. A (blue bar) indicates intra-examiner reliability for Examiner A; B (red bar), intra-examiner reliability for Examiner B; A&B (green bar), inter-examiner reliability. a. >2 mm of intra-examiner reliability for Examiner A. b. >2 mm of intra-examiner reliability for Examiner B. c. >2 mm of inter-examiner reliability. Landmarks were excluded if they satisfied even one of the criteria a, b, and c.

**Figure S3.** The inter- and intra-examiner reliability in the x- (top), y- (middle), and z- (bottom) axes for the mandibular surface. A (blue bar) indicates intra-examiner reliability for Examiner A; B (red bar), intra-examiner reliability for Examiner B; A&B (green bar), inter-examiner reliability. a. >2 mm of intra-examiner reliability for Examiner A. b. >2 mm of intra-examiner reliability for Examiner B. c. >2 mm of inter-examiner reliability. d. The same name landmarks as the landmarks showing >2 mm of intra-examiner reliability on the opposite side were deleted because we intended to employ a symmetrical landmark assignment. (For example, if the right anterior border of the ramus (ABR [#19]) showed greater errors, then we excluded the left ABR [#18].) The landmarks were excluded if they satisfied even one of the criteria a, b, c, and d.

**Figure S4.** 95% Confidence ellipse for the errors of the AI-identified landmarks (AI) when compared to the gold standard (GS).

**Figure S5-1.** Intraclass correlation (ICC) between mesh fitted based on the AI-identified landmarks (AI_MESH) and the gold standard (GS_MESH) in the maxilla. X, transverse direction; Y, vertical direction, Z, antero-posterior direction.

**Figure S5-2.** Intraclass correlation (ICC) between mesh fitted based on the AI-identified landmarks (AI_MESH) and the gold standard (GS_MESH) in the mandible. X, transverse direction; Y, vertical direction, Z, antero-posterior direction.
